# The effect of toll-like receptor ligands on energy metabolism and myokine expression and secretion in cultured human skeletal muscle cells

**DOI:** 10.1038/s41598-021-03730-w

**Published:** 2021-12-20

**Authors:** Ragna H. Tingstad, Frode Norheim, Fred Haugen, Yuan Z. Feng, Hege S. Tunsjø, G. Hege Thoresen, Arild C. Rustan, Colin Charnock, Vigdis Aas

**Affiliations:** 1 Department of Life Sciences and Health, Faculty of Health Sciences, Oslo Metropolitan University, Oslo, Norway; 2 Department of Nutrition, Institute of Basic Medical Sciences, University of Oslo, Oslo, Norway; 3 National Institute of Occupational Health, Oslo, Norway; 4 Section for Pharmacology and Pharmaceutical Biosciences, Department of Pharmacy, University of Oslo, Oslo, Norway; 5 Department of Pharmacology, Institute of Clinical Medicine, University of Oslo, Oslo, Norway

**Keywords:** Metabolism, Innate immunity

## Abstract

Skeletal muscle plays an important role in glycaemic control and metabolic homeostasis, making it a tissue of interest with respect to type 2 diabetes mellitus. The aim of the present study was to determine if ligands of Toll-like receptors (TLRs) could have an impact on energy metabolism and myokine expression and secretion in cultured human skeletal muscle cells. The myotubes expressed mRNA for TLRs 1–6. TLR3, TLR4, TLR5 and TLR6 ligands (TLRLs) increased glucose metabolism. Furthermore, TLR4L and TLR5L increased oleic acid metabolism. The metabolic effects of TLRLs were not evident until after at least 24 h pre-incubation of the cells and here the metabolic effects were more evident for the metabolism of glucose than oleic acid, with a shift towards effects on oleic acid metabolism after chronic exposure (168 h). However, the stimulatory effect of TLRLs on myokine expression and secretion was detected after only 6 h, where TLR3-6L stimulated secretion of interleukin-6 (IL-6). TLR5L also increased secretion of interleukin-8 (IL-8), while TLR6L also increased secretion of granulocyte–macrophage colony stimulating factor (GM-CSF). Pre-incubation of the myotubes with IL-6 for 24 h increased oleic acid oxidation but had no effect on glucose metabolism. Thus IL-6 did not mimic all the metabolic effects of the TLRLs, implying metabolic effects beyond the actions of this myokine.

## Introduction

In healthy human adults, skeletal muscle constitutes about 30–40% of the total body weight^1^. The metabolism of skeletal muscle is fuelled largely by carbohydrates and fatty acids, and it is a major insulin-sensitive organ, accounting for about 80% of the body’s insulin-stimulated glucose disposal^2,3^. Consequently, the critical role that skeletal muscle plays in glycaemic control and metabolic homeostasis makes it an organ of particular interest with regard to obesity and type 2 diabetes (T2D) mellitus.

Obesity and T2D have become major global health problems over the past few decades, particularly in younger age groups. The World Health Organization has reported that > 1.9 billion adults (above the age of 18), 340 million children and adolescents (5–19 years old) and 41 million children under the age of 5 have overweight or obesity^4,5^. In 2016, 422 million adults were suffering from diabetes^6^. It is well established that obesity is a risk factor for developing T2D, with > 80% of subjects with T2D being overweight^7^. In the words of Yach, Stuckler and Brownell: “Overweight and obesity have become to diabetes what tobacco is to lung cancer”^8^. In adipose tissue of individuals with obesity, macrophage infiltration with secretion of pro-inflammatory cytokines has been observed^9,10^, and although obesity now in its own right is considered to be a low grade, chronic inflammatory condition, the cause of this inflammation is still under some debate^11^.

It has been shown that gram-negative bacteria-derived lipopolysaccharide (LPS) levels in blood are elevated in individuals with obesity and T2D^12,13^, and that the plasma LPS concentration correlates negatively with muscle insulin sensitivity^14^. A single meal rich in fat elevated blood LPS in healthy subjects as well as in individuals with obesity and those with impaired glucose tolerance and T2D^15,16^. Amar and co-workers^17^ have also shown that following a week on a high-fat diet in mice, both adipose tissue and blood contained live commensals of the gut. Both gram-negative and gram-positive bacteria, such as members of the genus *Clostridium*, have been suggested to have a role in the development of T2D. Clostridia were, for example, found to be more abundant in subjects with T2D than in healthy human individuals^18^.

Bacterial components, known as pathogen-associated molecules (PAMPs), bind to pathogen recognition receptors (PRRs) of the innate immune system, in the host. One class of PRR is known as Toll-like receptors (TLRs). Upon activation by PAMPs, TLRs induce intra- and intercellular responses such as the release of pro-inflammatory cytokines, including interleukin-6 (IL-6), interleukin-8 (IL-8) and tumour necrosis factor (TNF), possibly contributing to the inflammation observed in obesity^19^.

Several lines of evidence suggest a role for TLRs in skeletal muscle energy metabolism, in particular TLR4 which is a receptor for both LPS^20^ and saturated fatty acids (palmitic acid), as demonstrated in both murine models^21–23^ and humans^24^. In murine skeletal muscle cells, exposure to LPS reduced insulin-stimulated phosphorylation of IRS-1, Akt and AS160, as well as insulin-stimulated glucose uptake. Furthermore, LPS induced transcription of pro-inflammatory cytokines, such as IL-6 and TNF^14,25^. These effects of LPS could be suppressed by the pharmacological TLR4 inhibitor TAK-242 and by TLR4 gene silencing^14,25^. It has also been shown that skeletal muscle cells from individuals with obesity and T2D express more TLR4 than lean controls and show indications of elevated NFkB/IkB-signalling^24^. With respect to TLRs other than TLR4, there has been a report of a negative effect of TLR3 on glucose uptake in skeletal muscle cells from pregnant women^26^. At present, little is known about the other TLRs and their effects on human skeletal muscle energy metabolism.

The aim of the present study was to determine if discrete TLR ligands could affect glucose and oleic acid metabolism, as well as myokine expression and secretion, in cultured human skeletal muscle cells.

## Materials and methods

### Materials

Dulbecco's Modified Eagle Medium (DMEM)-Glutamax low glucose, Foetal bovine serum (FBS), Amphotericin B, Penicillin/Streptomycin, Dulbecco's Phosphate-Buffered Saline (DPBS) (with CaCl_2_ and MgCl_2_) and Trypsin-Ethylenediaminetetraacetic acid (EDTA) were from Gibco (Gibco, Life technologies, Paisley, UK). Gentamicin, NaOH, oleic acid, glucose, Bovine Serum Albumin (BSA), L-carnitine, hydroxyethyl piperazineethanesulfonic acid (HEPES), cycloheximide, 96-well Corning CellBIND and Phosphate buffered saline (PBS) 10 × were from Sigma-Aldrich (St. Louis, MO, US). Insulin (100 IE/ml) was from Novo Nordisk (Bagsvaerd, Denmark). RNeasy Mini Kit and QuantiNova SYBR Green RT-PCR kit were from Qiagen (Düsseldorf, Germany). Ultroser G was from Pall (Cergy-Saint-Christophe, France). Human Toll-like receptor (TLR) 1–9 agonist kit, TLR1/2 Pam3CSK4 and TLR5 *S. typhimurium* flagellin were from InvivoGen (San Diego, CA, US). Toll-like Receptor ligands Set I was from Apotech (Epalinges, Switzerland). D-[U-^14^C]glucose (2.07 GBq/mmol) was from American Radiolabeled Chemicals (St. Louis, MO, US). [1-^14^C]oleic acid (2.01 GBq/mmol) was from PerkinElmer (Boston, MA, US). Bio-Rad Protein Assay Dye Reagent Concentrate, iScript cDNA synthesis kit and Bio-Plex System were from BioRad (Hercules, CA, US). OptiPhase Supermix, Unifilter-96 GF/B, 96-well Isoplate and TopSeal-A transparent film were from PerkinElmer (Shelton, CT, US). Human Cytokine Lincoplex Kit was from Millipore (Billerica, MA, US). Human RPLP0 endogenous control, the TaqMan Gene Expression Assay and the relative quantitation analysis module were from Applied Biosystems (ThermoFisher Scientific, Foster City, CA, US). Quibit RNA BR assay kit, primers for substrate transporter RT qPCR and Nunc EasYFlask (75cm^2^) Nunclon Delta Surface were from Thermo Scientific (ThermoFisher Scientific, Foster City, CA, US).

### Human skeletal muscle cell culture

Human satellite cells from *M. obliquus internus abdominis* of healthy donors were isolated as previously described by Gaster *et al*^27^. The donors (57% women) were 48 ± 13 (mean, SD) years of age with a body mass index of 23.8 ± 2.9 kg/m^2^ and a fasting glucose of 5.1 ± 0.5 mM. Plasma lipids and blood pressure were within the normal range and there was no family history of diabetes. The cells were proliferated on 96-well CellBind microplates in DMEM-Glutamax low glucose supplemented with 2% foetal bovine serum, 2% Ultroser G, penicillin (100 units/ml), streptomycin (100 µg/ml) and amphotericin B (1.25 µg/ml) for 5–7 days until 70–80% confluence, with morphology similar to as shown previously^28^. To induce cell differentiation from myoblasts to myotubes, the medium was changed to DMEM-Glutamax low glucose supplemented with 2% foetal bovine serum, penicillin (100 units/ml), streptomycin (100 µg/ml), amphotericin B (1.25 µg/ml) and insulin (25 pM). The cells were cultured in a humidified 5% CO_2_ atmosphere at 37 °C. Experiments were carried out 7 days after induction of differentiation.

### TLR ligand screening

Following differentiation, the multinucleated myotubes were exposed to 50 and 500 ng/ml Pam3CSK4 (TLR1/2L), 10^6^, 10^7^ and 10^8^ cells/ml heat-killed *Listeria monocytogenes* (HKLM) (TLR2L), 10 ng/ml, 1 and 50 µg/ml Poly (I:C) (TLR3L), 50 pg/ml, 10, 50, 100 and 500 ng/ml, 1, 2 and 10 µg/ml LPS (TLR4L), 50 pg/ml, 10 and 100 ng/ml flagellin (TLR5L) and 1 ng/ml and 1 µg/ml FSL-1 (TLR6L) (from the Human TLR 1–9 agonist kit from InvivoGen) in the differentiation media as described above prior to the substrate uptake and oxidation assays. The ligand concentrations were based on either the manufacturer’s suggested range or as previously described in published studies^29^.

### Substrate uptake and oxidation

The cells were exposed to ligands either during differentiation (chronic exposure – 168 h) or for 1, 3, 6 or 24 h prior to, or during (acute exposure) the substrate oxidation measurements. IL-6 was added to the cells 4 or 48 h prior to the substrate oxidation measurements. The energy substrates used were either D-[U-^14^C]glucose (37 kBq/ml, 200 µM) or [1-^14^C]oleic acid (37 kBq/ml, 100 µM). The energy substrates were added to DPBS with 20 mM HEPES. When using oleic acid, 1 mM L-carnitine was included in the substrate media to ensure maximum uptake of fatty acids by the mitochondria. The cells were washed with 37 °C DPBS before a 96-well Unifilter microplate soaked with 1 M NaOH (20 µl/well) was fixed on top of the cell plate as described previously by Wensaas *et al*^30^. The cells were incubated in a humidified 5% CO_2_ atmosphere at 37 °C for 4 h before the substrate-labelled medium was removed. The cells were washed twice with PBS at room temperature, lysed in 0.1 M NaOH and frozen at − 20 °C. The Unifilter containing trapped CO_2_ and the cell lysates (cell associated radioactivity, CA) were both counted by liquid scintillation with a PerkinElmer 2450 MicroBeta^2^ scintillation counter. Substrate uptake was calculated as the sum of trapped CO_2_ and cell associated radioactivity. Protein concentrations were measured with the BioRad protein assay according to the manufacturer’s protocol as described previously^31^ using a PerkinElmer VICTOR^3^ 1420 Multilabel counter. The substrate uptake and oxidation were calculated as nmol/mg protein. In order to inhibit protein synthesis following PAMP activation of the TLRs, 35 or 71 µM cycloheximide were added to the myotubes as described above, together with either LPS (10 or 50 ng/ml) or flagellin (10 or 50 ng/ml) 24 h before substrate uptake and oxidation were measured.

### Human cytokine LINCOplex protein expression

The human myotubes were cultured in 12-well cell culture plates as previously described by Haugen *et al*^32^ and exposed to 500 ng/ml Pam3CSK4 (TLR1/2L), 50 µg/ml Poly (I:C) (TLR3L), 1 µg/ml LPS (TLR4L), 100 ng/ml flagellin (TLR5L) and 100 ng/ml MALP-2 (TLR6L) (from the Toll-like receptor (TLR) ligand set 1 from Apotech) for 6 h. The conditioned media samples were collected for quantification of cytokines, quickly spun down to sediment any debris and analysed with the high sensitivity Human Cytokine LINCOplex Kit according to the manufacturer’s protocol, in duplicate samples. The microspheres targeting IL-6, IL-8 and GM-CSF were analysed by flow cytometry on a Bio-Plex System according to the manufacturer’s protocol. A standard curve of 0.13–2000 pg/ml was established for each myokine. For the samples, fluorescent readings for IL-6 and IL-8 fell within their respective standard curves. Control sample readings for GM-CSF were lower than the standard curve, and thus concentrations extrapolated by the software were included since the TLRL treated samples fell within the GM-CSF standard curve.

### Quantification of mRNA by real-time qPCR

For assessing TLR mRNA expression, total RNA from the human myotubes used for protein expression described above was isolated, reversely transcribed to cDNA and amplified using specific TaqMan Gene Expression Assays in a 96-well format, as previously described^32^. The following human target genes were monitored (the official gene symbol in parenthesis): Toll-like receptor 1 (TLR1), Hs00413978_m1; Toll-like receptor 2 (TLR2), Hs00610101_m1; Toll-like receptor 3 (TLR3), Hs00152933_m1; Toll-like receptor 4 (TLR4), Hs00152939_m1; Toll-like receptor 5 (TLR5), Hs00152825_m1; Toll-like receptor 6 (TLR6), Hs00271977_s1; Toll-like receptor 7 (TLR7), Hs00152971_m1; Toll-like receptor 8 (TLR8), Hs00152972_m1; Toll-like receptor 9 (TLR9), Hs00152973_m1. A pre-developed endogenous control targeting human large ribosomal protein P0 (RPLP0), Hs99999902_m1, was used to control for RNA loading and reverse transcription efficiency. The TLR mRNA expression levels were normalized against the RPLP0 control with the comparative C_T_ method for relative quantitation, as described in the manufacturer’s protocol.

For assessing the mRNA expression of substrate transporters (CD36, GLUT1 and GLUT4), human satellite cells were cultured in Nunc EasYFlask (75 cm^2^) Nunclon Delta Surface flasks as described above and exposed to either 100 ng/ml TLR4L or 100 ng/ml TLR5L, after 7 days of differentiation. The cells were harvested and total RNA from the cells was extracted with the Qiagen RNeasy mini kit as per the manufacturer’s protocol. RNA was quantified with the Qubit RNA Broad Range assay kit from ThermoFisher. The RNA samples were reversely transcribed to cDNA with the Bio Rad iScript cDNA synthesis kit as per the manufacturer’s protocol using an Applied Biosystems 2720 Thermal Cycler. Real time qPCR was carried out using the QuantiNova SYBR Green RT-PCR kit from Qiagen as described in the manufacturer’s protocol using a Stratagene MX3000p qPCR cycler. The following human target genes were monitored (with the official gene symbol in parenthesis): Platelet glycoprotein 4 (CD36), acc.no.: L06850, Solute carrier family 2 facilitated glucose transporter member 1 (SLC2A1, i.e., GLUT1), acc. no.: K03195, Solute carrier family 2 facilitated glucose transporter member 4 (SLC2A4, i.e., GLUT4) acc. no.: M20747 and glyceraldehyde-3-phosphate dehydrogenase (GAPDH), acc. no.: NM002046. GAPDH was analysed as a housekeeping gene and used for normalising the expression levels of CD36, SLC2A1 and SLC2A4.

### Statistical analysis

Comparisons of cytokine mRNA expression and secretion between ligand-treated groups and controls was performed by paired Student’s t-test. The level of significance was set to α = 0.05, and *p* < 0.05 was considered statistically significant. Linear mixed-model analysis (LMM, SPSS 26, IBM SPSS Inc., Chicago, IL, US) was used to calculate all metabolic effects of different treatment concentrations of TLRLs. Data in figures are given as means with single measurement values, represented by dots, unless otherwise stated and n = separate experiments. The number of biological parallels within each experiment are listed in all figure legends.

### Ethics

The study was conducted according to the guidelines of the Declaration of Helsinki and written and informed consent by the participants and approval by the Regional Committee for Medical and Health Research Ethics (Oslo, Norway, 2009/1095S-04133a) was obtained prior to the muscle biopsy sampling.

## Results

### TLR mRNA expression

**Figure 1:**
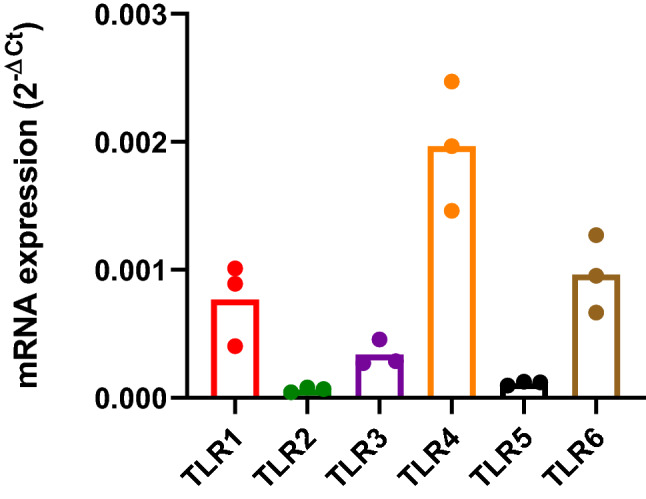
mRNA expression of toll like receptors (TLRs) in human myotubes. Myotubes were grown in 12-well plates, and the mRNA expression of TLRs 1–9 was measured by real time qPCR and normalized against the endogenous control targeting human large ribosomal protein P0 (RPLP0). Data are shown as means, with single measurement points (*n* = 3). TLRs 7–9 were undetectable.

Expression of TLR mRNA in human myotubes established from the *M. obliquus internus abdominis* was performed by real time qPCR. We observed that the cells expressed TLRs 1–6 (Fig. 1), while there was undetectable expression of TLRs 7, 8 and 9.

### Myokine expression and secretion following exposure to TLR ligands

**Figure 2:**
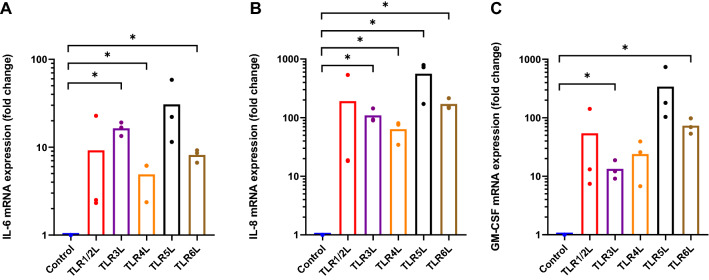
Myokine mRNA expression after exposure to TLR ligands. Human myotubes were cultured in 12-well CellBind plates and exposed to TLR1/2L (Pam3CSK4 500 ng/ml), TLR3L (Poly (I:C) 50 µg/ml), TLR4L (LPS 1 µg/ml), TLR5L (flagellin 100 ng/ml), and TLR6L (MALP-2 100 ng/ml) for 6 h, and mRNA was measured by real time qPCR of (**A**) IL-6, (**B**) IL8, and (**C**) GM-CSF. Data are shown as means, with single measurement points (*n* = 3). *p < 0.05 versus control by paired Student’s t-test. Control = 1.

Functionality of the TLRs was assessed by the ability of TLRLs to stimulate mRNA expression and secretion of pro-inflammatory myokines. Following 6 h of exposure to TLRLs, mRNA expression of the myokines IL-6, IL-8 and GM-CSF was assayed (Fig. 2). TLR3L and TLR6L significantly increased expression of all three myokines (Fig. 2A-C), whereas TLR4L increased IL-6 and IL-8 (Fig. 2A, B), and TLR5L increased IL-8 mRNA expression (Fig. 2B).

**Figure 3:**
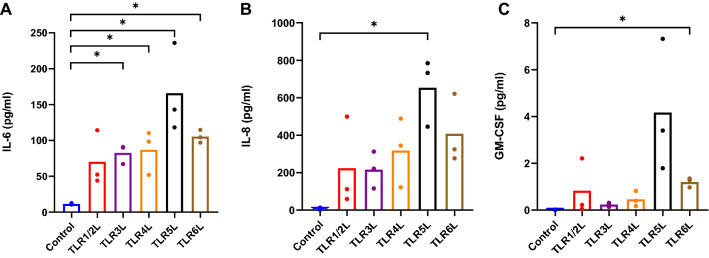
Myokine secretion following exposure of myotubes to TLR ligands. Human myotubes were cultured in 12-well CellBind plates and exposed to TLR1/2L (Pam3CSK4 500 ng/ml), TLR3L (Poly (I:C) 50 µg/ml), TLR4L (LPS 1 µg/ml), TLR5L (flagellin 100 ng/ml) and TLR6L (MALP-2 100 ng/ml) for 6 h. The cell media were collected following ligand exposure, and myokine secretion was measured with the high sensitivity human cytokine LINCOplex kit. (**A**) Interleukin-6 (IL-6) secretion (pg/ml), control: 11.5 ± 0.6 pg/ml, (**B**) Interleukin-8 (IL-8) secretion (pg/ml), control: 6.7 ± 3.5 pg/ml and (**C**) Granulocyte–macrophage colony-stimulating factor (GM-CSF) secretion (pg/ml), control: 0.01 ± 0.01 pg/ml. The GM-CSF control is based on extrapolation of the standard curve. Data are shown as means, with single measurement points (*n* = 3). *p < 0.05 vs control by paired Student’s t-test.

We also wanted to see whether the myokines were secreted, which would enable paracrine and/or autocrine effects. The secretion of myokines to the cell media was assessed with the high sensitivity Human Cytokine Lincoplex Kit (Fig. 3). TLR3L and TLR4L significantly increased secretion of IL-6 (Fig. 3A). TLR5L increased both IL-6 and IL-8 secretion (Fig. 3A, B), whereas TLR6L increased secretion of IL-6 and GM-CSF (Fig. 3A, C).

### The effect of TLR ligands on glucose and fatty acid metabolism in human myotubes

**Figure 4:**
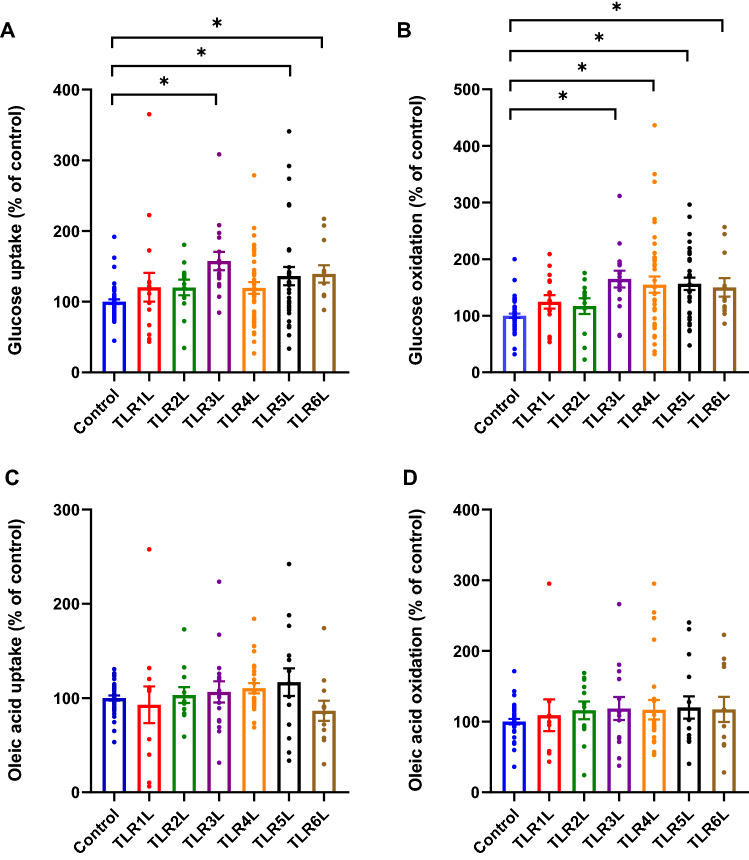
Effects of TLR ligands on uptake and oxidation of glucose and oleic acid in human myotubes. Human myotubes were grown in 96-well CellBind microplates and exposed to TLR1/2L (Pam3CSK4 50 ng/ml), TLR2L (HKLM 10^7^ cells/ml), TLR3L (Poly (I:C) 10 ng/ml), TLR4L (LPS 100 ng/ml), TLR5L (flagellin 10 ng/ml), and TLR6L (FSL-1 1 ng/ml) for 24 h prior to incubation with either D-[U-^14^C]glucose (37 kBq/ml, 200 µM) or [1-^14^C]oleic acid (37 kBq/ml, 100 µM). (**A**) Uptake of glucose was assessed as the sum of oxidized D-[U-^14^C]glucose (CO_2_) and the remaining cell-associated radioactivity. (**B**) Oxidized D-[U-^14^C]glucose (CO_2_) was trapped in a filter and counted by liquid scintillation. Data are given as % of respective controls as mean, with single measurement points. *n* = 3–10 individual experiments, each experiment with 4 parallels per ligand and concentration. The average glucose uptake was 29.7 ± 6.0 nmol/mg protein and glucose oxidation was 17.4 ± 4.8 nmol/mg protein in control cells. (**C**) Uptake of oleic acid was assessed as the sum of oxidized [1-^14^C]oleic acid (CO_2_) and the remaining cell-associated radioactivity (CA). (**D**) Oxidized [1-^14^C]oleic acid (CO_2_) was trapped in a filter and counted by liquid scintillation. Data are given as % of respective controls as mean, with single measurement points. *n* = 3–6 individual experiments, each experiment with 4 parallels per ligand and concentration. The average oleic acid uptake was 67.4 ± 10.4 nmol/mg protein in control cells and oleic acid oxidation was 6.2 ± 1.7 nmol/mg protein. *p < 0.05 vs control (Linear Mixed Model, SPSS).

To evaluate whether TLRLs might be involved in metabolic regulation or affect energy metabolism in skeletal muscle cells, human myotubes were pre-incubated with TLR1/2L (Pam3CSK4 50 ng/ml), TLR2L (HKLM 10^7^ cells/ml), TLR3L (Poly (I:C) 10 ng/ml), TLR4L (LPS 100 ng/ml), TLR5L (flagellin 10 ng/ml), and TLR6L (FSL-1 1 ng/ml) for 24 h before glucose and oleic acid uptake and oxidation were measured (Fig. 4). TLR3L, TLR5L and TLR6L all increased glucose uptake by 57% (SEM ± 13%), 36% (SEM ± 13%) and 39% (SEM ± 12%), respectively (Fig. 4A). TLR4L also seemed to increase glucose uptake (*p* = 0.083), although not significantly. TLR3L, TLR4L, TLR5L and TLR6L increased glucose oxidation by 65% (SEM ± 15%), 55% (SEM ± 14%), 57% (SEM ± 11%) and 50% (SEM ± 16%), respectively (Fig. 4B). There were no observed statistically significant changes in metabolism of oleic acid (Fig. 4C, D).

**Figure 5:**
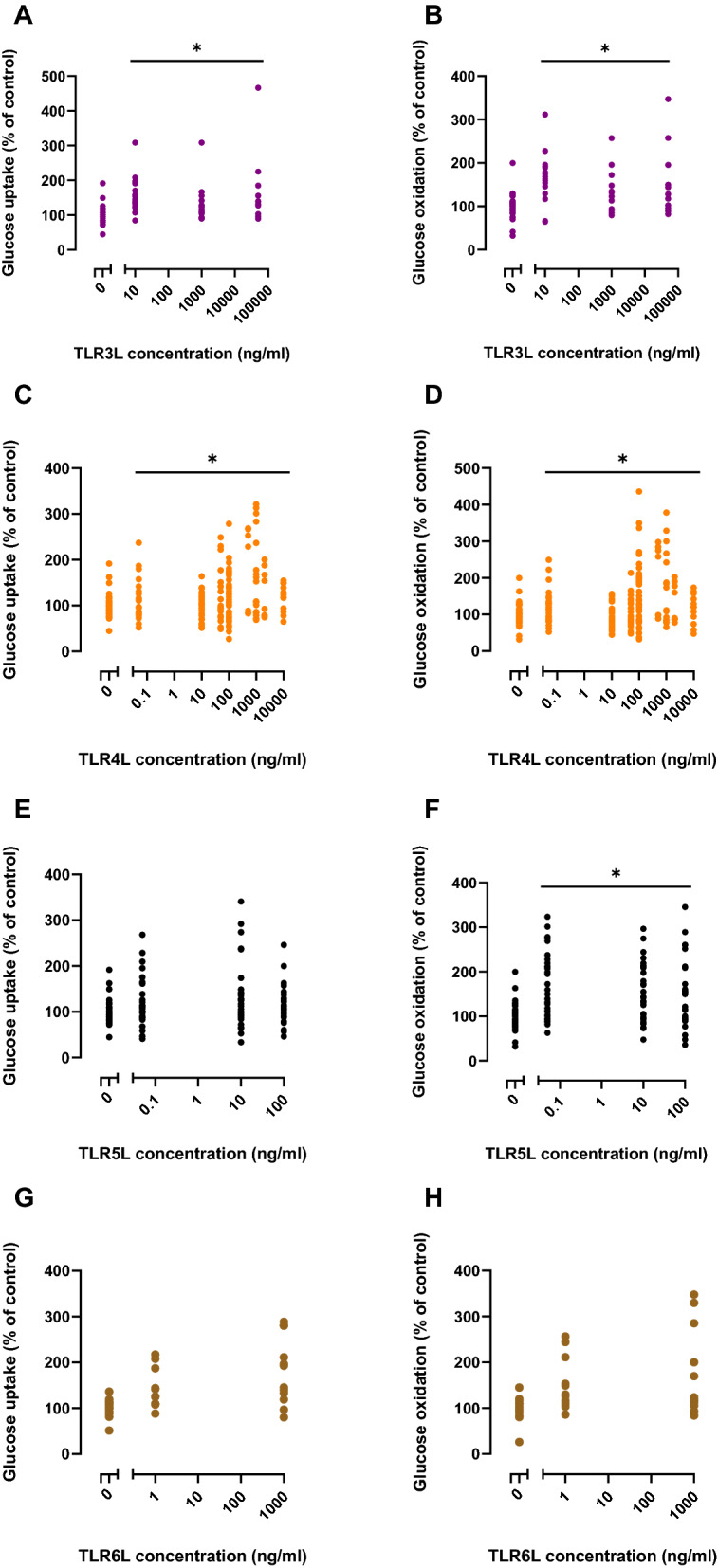
Concentration–response effects of TLR ligands (TLRLs) on glucose uptake and oxidation in human myotubes. Human myotubes were grown in 96-well CellBind microplates and exposed to TLR3L (Poly (I:C) 10, 1000 and 50,000 ng/ml), TLR4L (LPS 0.05, 10, 50, 100, 500, 1000, 2000 and 10,000 ng/ml), TLR5L (flagellin 0.05, 10 and 100 ng/ml), and TLR6L (FSL-1 1 and 1000 ng/ml) for 24 h prior to incubation with D-[U-^14^C]glucose (37 kBq/ml, 200 µM). (**A**), (**C**), (**E**), (**G**) Uptake of glucose was assessed as the sum of oxidized D-[U-^14^C]glucose (CO_2_) and the remaining cell-associated radioactivity. (**B**), (**D**), (**F**), (**H**) Oxidized D-[U-^14^C]glucose (CO_2_) was trapped in a filter and counted by liquid scintillation. Data are given as % of respective controls. All single data points from 3–10 individual experiments are shown. The average glucose uptake was 29.7 ± 6.0 nmol/mg protein and glucose oxidation was 17.4 ± 4.8 nmol/mg protein in control cells. *p < 0.05 vs control, overall effect (Linear Mixed Model, SPSS).

**Figure 6:**
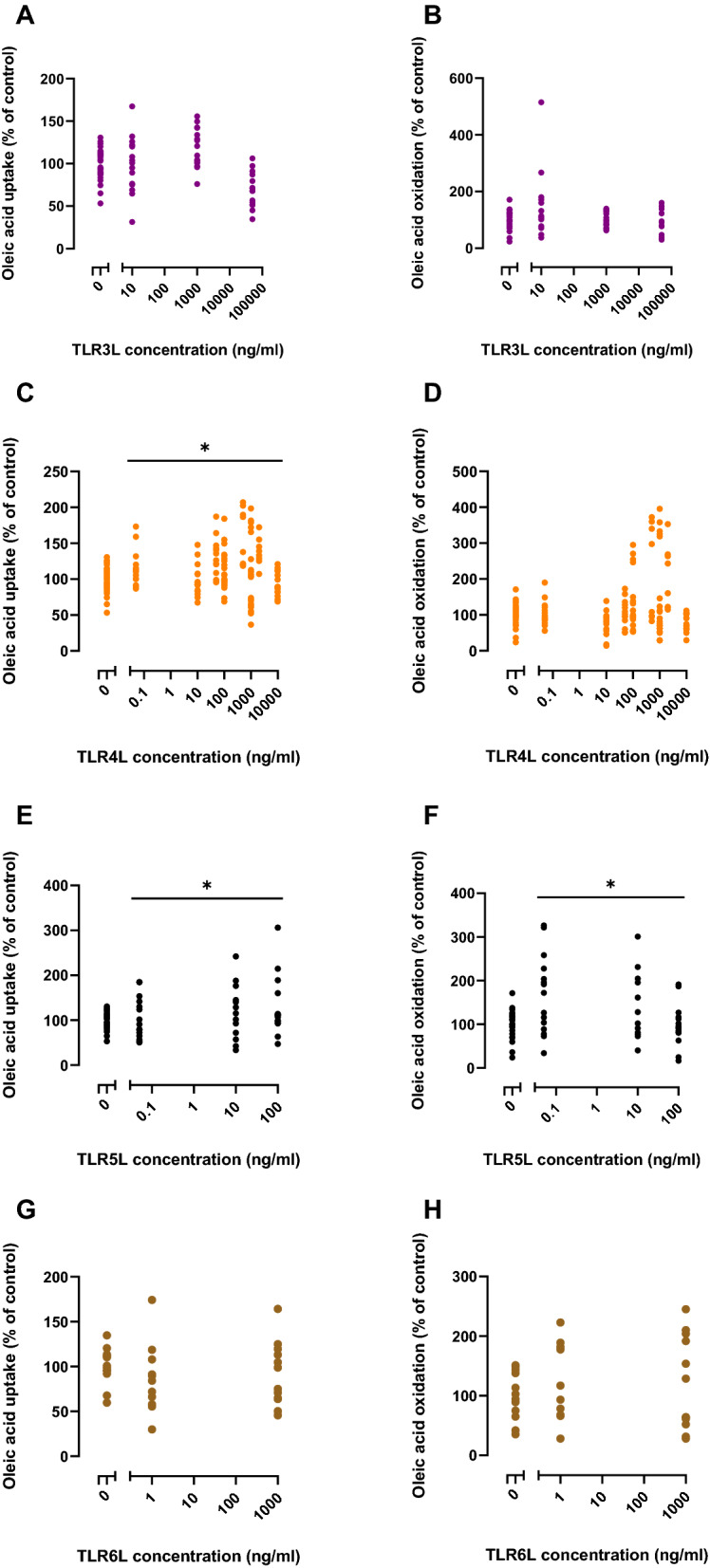
Concentration–response effects of TLR ligands (TLRLs) on oleic uptake and oxidation in human myotubes. Human myotubes were grown in 96-well CellBind microplates and exposed to TLR3L (Poly (I:C) 10, 1000 and 50,000 ng/ml), TLR4L (LPS 0.05, 10, 50, 100, 500, 1000, 2000 and 10,000 ng/ml), TLR5L (flagellin 0.05, 10 and 100 ng/ml), and TLR6L (FSL-1 1 and 1000 ng/ml) for 24 h prior to incubation with [1-^14^C]oleic acid (37 kBq/ml, 100 µM). (**A**), (**C**), (**E**), (**G**) Uptake of oleic acid was assessed as the sum of oxidized [1-^14^C]oleic acid (CO_2_) and the remaining cell-associated radioactivity (CA). (**B**), (**D**), (**F**), (**H**) Oxidized [1-^14^C]oleic acid (CO_2_) was trapped in a filter and counted by liquid scintillation. Data are given as % of respective controls. All single data points from 3–6 individual experiments are shown. The average oleic acid uptake was 67.4 ± 10.4 nmol/mg protein and oleic acid oxidation was 6.2 ± 1.7 nmol/mg protein in control cells. *p < 0.05 vs control, overall effect (Linear Mixed Model, SPSS).

Concentration-dependency of the TLRLs on glucose (Fig. 5) and oleic acid (Fig. 6) metabolism was examined after 24 h of exposure. Statistical analysis shows overall effects calculated by Linear Mixed Model. Compared to the control, TLR3L and TLR4L significantly increased glucose uptake by 49 and 24%, respectively on average (Fig. 5A, C). TLR3L, TLR4L and TLR5L increased glucose oxidation by 49, 33 and 58%, respectively (Fig. 5B, D, F). In these experiments, TLR4L and TLR5L also affected oleic acid metabolism (Fig. 6) and increased oleic acid uptake by about 13 and 15%, respectively (Fig. 6C, E). However, oleic acid oxidation was significantly increased by 31% only for TLR5L (Fig. 6F).

### Time-course effects of TLR ligands on glucose and fatty acid metabolism in human myotubes

**Figure 7:**
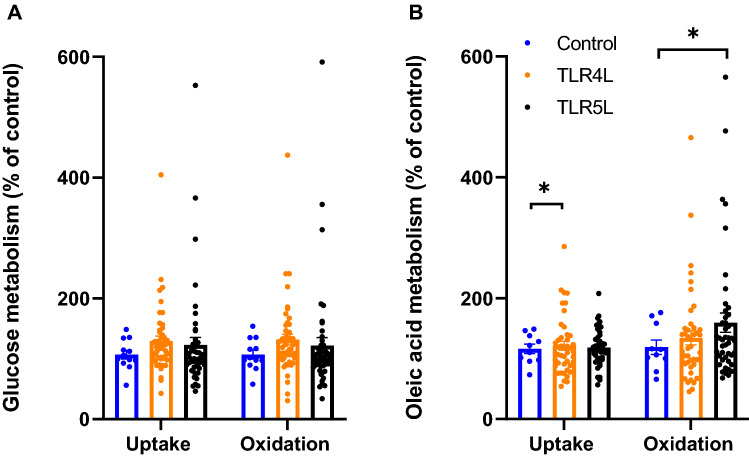
Overall effects of chronic exposure of human myotubes to TLR4L and TLR5L on glucose and fatty acid metabolism. Human myotubes were grown in 96-well CellBind microplates and exposed to TLR4L (LPS, 0.05–100 ng/ml) or TLR5L (flagellin, 0.05–100 ng/ml) for 168 h (chronic) prior to 4 h of incubation with D-[U-^14^C]glucose (37 kBq/ml, 200 µM) or [1-^14^C]oleic acid (37 kBq/ml, 100 µM). (**A**) Oxidized D-[U-^14^C]glucose (CO_2_) was trapped in a filter and counted by liquid scintillation, and uptake of substrate was assessed as the sum of oxidized (CO_2_) and the remaining cell-associated radioactivity (CA). (**B**) Oxidized [1-^14^C]oleic acid (CO_2_) was trapped in a filter and counted by liquid scintillation, and uptake of substrate was assessed as the sum of oxidized (CO_2_) and the remaining cell-associated radioactivity (CA). Data are given as means and all single data points are shown (*n* = 3 individual experiments, each experiment with 4 parallels per ligand). *p < 0.05 vs control, overall effect (Linear Mixed Model, SPSS).

To investigate whether shorter or longer durations of ligand exposure had an impact on energy metabolism, a time-course experiment was carried out with TLR4L and TLR5L. The cells were exposed to the ligands either acutely during the energy substrate assay (acute exposure of 4 h), or for 1, 3, 6 or 168 h (chronic exposure) prior to the assay. None of the short time exposures (acute during assay or 1–6 h pre-treatment) induced changes in glucose or oleic acid metabolism (data not shown). After extended exposure (chronic, 168 h) to TLR4L, both uptake and oxidation of glucose seemed increased, as found after 24 h (Fig. 4), though the response was not statistically significant (Fig. 7A, *p* = 0.07 for both). The overall effects of TLR4L and TLR5L on increasing oleic acid metabolism were however preserved after 168 h, with TLR4L increasing oleic acid uptake and TLR5L increasing oleic acid oxidation by 19 and 57%, respectively (Fig. 7B). The effect of TLR5L on oleic acid uptake and the effect of TLR4L on oleic acid oxidation also seemed to be increased, although not significantly (*p* = 0.07 and *p* = 0.06, respectively).

### Effect of TLR ligands on substrate transporter expression in human myotubes

**Figure 8:**
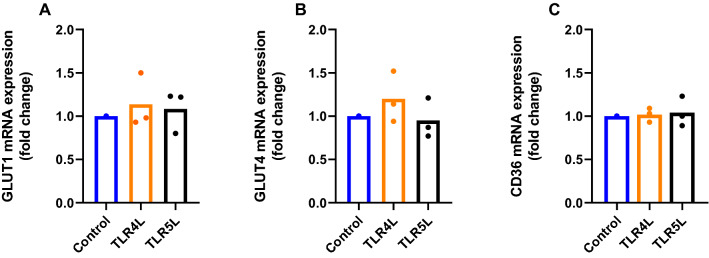
mRNA expression of substrate transporters in human myotubes. Myotubes were grown in Nunc EasYFlask (75 cm^2^) Nunclon Delta Surface flasks and exposed to either TLR4L (100 ng/ml LPS) or TLR5L (100 ng/ml flagellin) for 24 h. The mRNA expression of the glucose transporters (**A**) GLUT1 (SLC2A1) and (**B**) GLUT4 (SLC2A4) or (**C**) the fatty acid transporter CD36 was measured by real time qPCR and normalized against the endogenous control targeting glyceraldehyde-3-phosphate dehydrogenase (GAPDH). Data are shown as means with all single measurements shown (*n* = 3).

Given the changes observed in glucose and oleic acid metabolism following exposure to TLRLs, we wanted to assess if these effects could be due to alterations in the expression levels of the glucose transporters GLUT1 (SLC2A1) and GLUT4 (SLC2A4) or the fatty acid transporter CD36 (also known as platelet glycoprotein 4), following 24 h exposure of human myotubes to either TLR4L or TLR5L. Using real time qPCR to quantify the transporter mRNA expression, we detected no ligand-induced changes in the expression levels of these substrate transporters (Fig. 8).

### Effect of interleukin-6 (IL-6) on glucose and oleic acid metabolism in human myotubes

**Figure 9:**
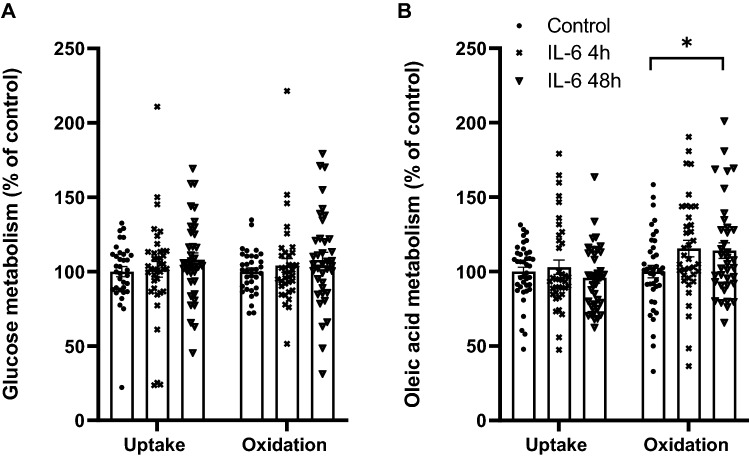
Effect of interleukin-6 (IL-6) on glucose and oleic acid metabolism in human myotubes. Human myotubes were grown in 96-well CellBind microplates and exposed to 10 ng/ml interleukin-6 for 4 h during or 48 h prior to incubation with D-[U-^14^C]glucose (37 kBq/ml, 200 µM) or [1-^14^C]oleic acid (37 kBq/ml, 100 µM). (**A**) Uptake and oxidation of glucose. (**B**) Uptake and oxidation of oleic acid. Oxidized substrate was trapped in a filter and counted by liquid scintillation, and uptake of substrate was assessed as the sum of oxidized (CO_2_) and the remaining cell-associated radioactivity (CA). Glucose uptake and oxidation in control cells were 37.6 ± 2.8 nmol/mg protein and 31.6 ± 2.1 nmol/mg protein, respectively. Oleic acid uptake and oxidation were 75.2 ± 13.8 nmol/mg protein and 10.7 ± 0.6 nmol/mg protein, respectively. Data are presented as mean with all single measurements shown (*n* = 5 individual experiments, each experiment with 4 parallels). *p < 0.05 vs control (Linear Mixed Model, SPSS).

TLRLs were shown to increase myokine production and secretion already after 6 h of exposure, whereas metabolic effects were first seen after 24 h. Possibly, the metabolic effects of TLRLs could be mediated indirectly by the secreted myokines. To test this, we first assessed the metabolic effects of TLR4L and TLR5L in the presence of the protein synthesis inhibitor cycloheximide. Cycloheximide did not quench these effects (data not shown). Moreover, we wanted to compare the effects of IL-6 on myotube energy metabolism to the effects mediated by TLRLs, in order to investigate if IL-6 could mimic the effects of the TLRLs. Myotubes were incubated with IL-6 for 4 h during substrate trapping or for 48 h prior to substrate trapping (Fig. 9A, B). IL-6 had no significant effect on glucose metabolism (Fig. 9A), whereas it increased oleic acid oxidation after 48 h (Fig. 9B), by 14%. This suggests that some, but not all the effects mediated by TLRLs might be mimicked by IL-6.

## Discussion

In this study we have examined whether TLR1-6 ligands might affect energy metabolism in primary human myotubes. We have shown that TLRs 1–6 are expressed in cultured human myotubes, and furthermore, their functionality was confirmed by both myokine mRNA expression and secretion, and treatment with specific ligands for several of the TLRs had effects on both glucose and oleic acid metabolism.

We were able to show that cultured human myotubes established from the *M. obliquus internus abdominis* expressed TLRs 1–6. However, there was undetectable expression levels of TLRs 7, 8 and 9. By comparison, mRNAs for TLRs 1–10 have previously been reported in human skeletal muscle biopsies^33^, whereas in C2C12 cells and mouse skeletal muscle TLRs 1–7 were expressed^34^. It has been established that TLR4 in particular is both present and active in human skeletal muscle, and observations have shown increased expression in both subjects with obesity and/or T2D^21,22,24^. In the present study, we have completed a screen of recognised TLRLs on human skeletal muscle cell functions, measuring myokine expression and secretion as well as metabolic functions. The results showed that exposing myotubes to TLR3-6L resulted in increased expression of myokine mRNA, as well as increased myokine secretion and enhanced cellular energy metabolism, particularly glucose metabolism.

IL-6 is the most well-known myokine. We found that the mRNA expression levels of IL-6 were increased following exposure to TLR3L, -4L and -6L, and that exposure to these ligands, in addition to TLR5L, also gave rise to an increase in IL-6 secretion. Previous studies have shown that TLR4L increased IL-6 expression and secretion from skeletal muscle in murine models, as well as in cell lines of murine and human origin^34–36^. In line with our work, the LPS concentrations used in these studies ranged from 50 pg/ml to 10 µg/ml, and the exposure times were comparable.

IL-8 mRNA expression in our study was increased following exposure to TLR3-6L, but only TLR5L increased secretion of IL-8. TLR3L has previously been shown to increase gene expression of both IL-6 and IL-8 in skeletal muscle biopsies from pregnant women^26^. However, to our knowledge, there is no information available on the possible effects of TLRLs on mRNA expression and secretion of IL-8 in cultured human skeletal muscle cells.

We also report that GM-CSF mRNA expression increased following exposure to TLR3L and TLR6L, and TLR6L also increased GM-CSF secretion. GM-CSF has been observed to be secreted from a number of different cell types in vitro upon exposure to pro-inflammatory stimuli such as LPS^37^, and it has been shown that the cardiotonic steroid ouabain promoted secretion of GM-CSF in human skeletal muscle cells in both healthy subjects and those with T2D^38^. To our knowledge, the work presented here is the first showing the expression and secretion of GM-CSF by cultured human myotubes following exposure to TLR6L and TLR3L. The GM-CSF values of the control sample fell below the established standard curve, and therefore the values had to be extrapolated by the software. It can then be assumed that under normal conditions, human skeletal muscle cells do not secrete great quantities of GM-CSF. However, all the TLRL treated samples had higher GM-CSF levels. Our findings suggest that human skeletal muscle can contribute to systemic inflammation and given the sheer proportion of body mass muscles represent, skeletal muscle continues to be an organ of clinical interest in chronic inflammation.

We have further shown that some of the TLRLs affected metabolism of glucose and oleic acid in cultured human myotubes. These effects were more evident for the metabolism of glucose than oleic acid following 24 h of ligand exposure, with a shift towards effects on oleic acid metabolism after chronic exposure.

Exposure of the myotubes to TLR3-6L (Poly (I:C) 10 ng/ml, LPS 100 ng/ml, flagellin 10 ng/ml, and FSL-1 1 ng/ml, respectively) for 24 h increased both glucose uptake and oxidation but had no effect on oleic acid metabolism. With increasing concentrations and calculating the overall effects, TLR4L (LPS up to 10,000 ng/ml) increased oleic acid uptake, and TLR5L (flagellin up to 100 ng/ml) increased both oleic acid uptake and oxidation. The effects of TLRLs on glucose metabolism were transient, whereas both TLR4L and TLR5L caused a trend toward both increased uptake and oxidation of oleic acid after ligand exposure for the entire differentiation period (168 h treatment). However, the only statistically significant changes found after chronic exposure were that TLR4L increased oleic acid uptake and TLR5L increased oleic acid oxidation.

There is previous evidence of an increase in glucose utilisation in human primary myotubes exposed to TLR4L^35^. However, in contrast to our findings, the same researchers also observed a decrease in fatty acid oxidation. It should also be noted that palmitic acid was used in that study, and previous work has suggested that oleic and palmitic acid are handled differently in skeletal muscle cells^39^. Thus, the effect of LPS on palmitic acid and oleic acid metabolism might differ. In stark contrast to our findings, it has been reported that TLR3L caused a decrease in glucose uptake in skeletal muscle biopsies from pregnant women^26^. However, the muscle tissue preparation in this latter study is not directly comparable to our cell culture model, and this might explain the contradictory results.

In the concentration ranges used, neither TLR3L (Poly (I:C) 10–50,000 ng/ml), TLR4L (LPS 0.05–10,000 ng/ml), TLR5L (flagellin 0.05–100 ng/ml) nor TLR6L (FSL-1 1–1000 ng/ml) showed concentration-dependent effects on metabolism. The concentrations used in this study are largely based on the suggested range provided by the manufacturer^40^, as there is limited documentation on observed concentrations in vivo for the TLRLs other than TLR4L (LPS). In previous studies investigating metabolic endotoxemia^20,41,42^, LPS concentrations have been measured to be in the pg/ml range. Thus, the concentrations of the ligands used in this study might be higher than the expected in vivo concentrations, and the lack of concentration-dependency might be explained by the use of concentrations higher than what gives the maximal effects. Also, in another study reporting effects of TLR4L on glucose metabolism in human myotubes, the effects reported were independent of concentration (50 pg/ml and 500 ng/ml of LPS)^35^. In addition, as the effects of TLRLs on metabolism are small, and the biological variability is relatively large, it might be difficult to find significant concentration-dependency relationships. A possible explanation for the modest changes observed could stem from the limitations in using a cellular model. For example, there might be a requirement of other co-factors or physiological components to elicit greater metabolic alterations than observed in this study.

It has previously been shown that donor age affects both glucose and oleic acid metabolism in cultured skeletal muscle cells by decreasing oxidation of both substrates^43^. Here, however, we have presented an increase in both glucose and oleic acid oxidation following TLRL exposure, and it is possible to hypothesise that we might have observed greater changes in myotubes established from a younger set of donors.

Since both glucose and oleic acid uptake were increased after exposure to TLR4L and TLR5L, we hypothesised that expression of the substrate transporters might be increased. These human myotubes have previously been shown to express GLUT1, GLUT4 and CD36^44,45^, but following 24 h of TLR4L and TLR5L exposure, no changes in the mRNA expression of these substrate transporters was found. We can, therefore, not attribute the observed increased substrate uptake to an upregulation of mRNA expression of these transporters following exposure to these TLRLs.

Whereas the myokine secretion was increased only 6 h after addition of TLRLs, the metabolic effects were not evident until after 24 h. We therefore hypothesised that autocrine signalling of myokines could explain some of the metabolic effects of TLRLs. To test this, we added the TLRLs together with the protein synthesis inhibitor cycloheximide, and then measured glucose and oleic acid metabolism. Inhibition of protein synthesis by cycloheximide has the drawback of broadly targeting all protein synthesis, with major consequences for cellular functions. Thus, some caution is required in the interpretation of the observed effects. However, the metabolic effects of TLR4L and TLR5L were not abolished by cycloheximide, implying a more directly mediated effect. Moreover, exposure to IL-6 (10 ng/ml) increased oxidation of oleic acid in human myotubes after 48 h of exposure, although to a lesser extent than the TLRLs. Although we found that exposure to the TLR3L, -4L, -5L and -6L all gave rise to an increase in IL-6 secretion, we did not observe any changes in oleic acid uptake by all these ligands. Further, the effect of these ligands on glucose metabolism was not mimicked by IL-6. This effect of IL-6 is in line with previous findings, where it has been observed that infusion of recombinant human IL-6 in healthy male subjects gave rise to an increase in systemic fatty acid oxidation, but without observed changes in glucose metabolism^46^. Another study has also shown that when myotubes differentiated from myoblasts isolated from the *vastus lateralis* muscle from healthy subjects were exposed to IL-6 (25 ng/ml for 1 h) fatty acid oxidation increased, though in contrast to our findings, these researchers also observed an increase in glucose uptake^47^. As we have only investigated the effects of IL-6, our results cannot exclude the possible involvement of other myokines in the altered energy metabolism observed in human skeletal muscle cells. Further studies should be performed to investigate if the effects observed extend to other myokines.

A premise for the work we have presented within this article is the access of microbial components, whether gut bacteria or their products, to the blood stream and thereby skeletal muscle. The gut microbiota as a driving force of chronic inflammation has indeed received much attention in recent years^48,49^. It is enticing to hypothesise that the gut microbiota could give rise to such metabolic alterations that we have presented within this study. Although the connection between altered microbiota and obesity remains a highly complex issue, it is clear that changes in microbiota composition has the potential to play a part in the development of obesity and subsequent T2D^50^.

While the TLRL concentrations we have used in this study for most part are greater than physiological concentrations, we believe that taken together, our results show a proof of concept that TLRLs can exert effects on skeletal muscle metabolism. We show that IL-6 and TLR5L have similar effects on human skeletal muscle cells, as they both increase oleic acid oxidation. Where IL-6 showed this effect after 48 h, the TLR5L showed it after 24 h and preserved it after chronic (168 h) exposure. We have also presented other effects of the TLRLs on energy metabolism in human myotubes, though we cannot exclude that these effects may also be partly due to secretion of IL-6 or other myokines. The presence of the TLRs and the metabolic effects observed indicate that ligands of TLRs may indeed play a role in altering energy metabolism in human myotubes.

## Conclusions

In conclusion, we have shown that the TLR receptors 1–6 are expressed in cultured human myotubes isolated from the *M. obliquus internus abdominis*. Following exposure to TLRLs, we have demonstrated functionality of the receptors by increased mRNA expression and secretion of several pro-inflammatory myokines. In addition, we have shown that TLR3-6L can affect glucose metabolism and TLR4L and TLR5L oleic acid metabolism in cultured human myotubes. Further work is required to assess if these effects might be direct or mediated by TLR-induced myokine secretion. However, based on our results an indirect effect mediated by autocrine action of myokines, such as IL-6, cannot be ruled out, particularly on oleic acid metabolism.
